# Super-resolution binding activated localization microscopy through reversible change of DNA conformation

**DOI:** 10.1080/19491034.2017.1419846

**Published:** 2018-03-01

**Authors:** Aleksander Szczurek, Udo Birk, Hans Knecht, Jurek Dobrucki, Sabine Mai, Christoph Cremer

**Affiliations:** a Institute of Molecular Biology, Mainz, Germany; b Department of Cell Biophysics, Faculty of Biochemistry, Biophysics and Biotechnology, Jagiellonian University, Krakow, Poland; c Physics Department University of Mainz (JGU), Mainz, Germany; d McGill, Jewish General Hospital, Montreal, Quebec, Canada; e University of Manitoba, Cancer Care Manitoba, Winnipeg, Canada; f Institute of Pharmacy and Molecular Biotechnology (IPMB), Heidelberg University, Germany

**Keywords:** chromatin, DNA, fBALM, SMLM, nucleus, single molecule localization, super-resolution microscopy

## Abstract

Methods of super-resolving light microscopy (SRM) have found an exponentially growing range of applications in cell biology, including nuclear structure analyses. Recent developments have proven that Single Molecule Localization Microscopy (SMLM), a type of SRM, is particularly useful for enhanced spatial analysis of the cell nucleus due to its highest resolving capability combined with very specific fluorescent labeling. In this commentary we offer a brief review of the latest methodological development in the field of SMLM of chromatin designated DNA Structure Fluctuation Assisted Binding Activated Localization Microscopy (abbreviated as fBALM) as well as its potential future applications in biology and medicine.

## Chromatin structure investigation constitutes a challenge

The discovery that the genetic information within the DNA is organized as a double helix has revolutionized the understanding of its duplication and heredity. This finding alone, however, did not account for how the DNA with a total length of approximately ∼2 m can fold in order to fit into a human cell nucleus of a diameter 5 orders of magnitude smaller. In order for this to happen, the formation of chromatin, a complex of DNA and proteins, is necessary. The DNA is wrapped around histone protein octamers to form nucleosomes, having on average 146 base pairs (bp), with a linker DNA ranging from 10 to 50 bp in between the nucleosome complexes. However, at a higher level of packaging and condensation the precise higher order spatial organization of the chromatin is still a subject to an ongoing debate [1,2]. After mitosis, condensed chromosomes undergo decondensation and the nuclei of daughter cells are formed. Here, they form rather distinct chromosome territories and typically maintain their radial position in the nucleus throughout the cell cycle [3].

Many models of chromatin organization and regulation have been discussed previously [4–6]. Using chromosome conformation capture methods [7], it has been suggested that the genome is divided into two nuclear compartments: compartment A is enriched in active genes whereas compartment B comprises sequences of limited transcriptional potency [8,9]. Moreover, this technology has indicated the existence of small self-interacting chromatin domains, denoted as “topologically associated chromatin domains” (TADs). TADs are genomic regions of kilo- to mega- base-pairs that undergo a high frequency of local contacts while interacting infrequently with sequences within adjacent TADs [8]. In turn, using microscopic approaches, the DNA was demonstrated to have a very heterogeneous distribution across the cell nucleus with some regions essentially depleted of it (reviewed in [1]). A- and B- nuclear compartments were later demonstrated to spatially segregate and occupy the interior or the periphery of the cell nucleus, respectively [10]. Nonetheless, the chromatin architecture at an intermediate length-scale between a chromatin fiber and TADs (10 – 200 nm) remains elusive. Furthermore, the understanding of how the chromatin structure responds to various pathological situations and malfunctions is still limited. This includes different conditions such as Alzheimer's Disease [11], senescence [12], cancer [13], ischemia [14], and others. Hence the importance to develop appropriate methodology able to visualize tangled and complex chromatin arrangements at enhanced resolution.

## Super-resolution microscopy as a potent tool in chromatin structure analysis

Super-Resolution Microscopy (SRM) is a family of fluorescence-based methods that have enabled studying biological structures (mostly in cell culture samples) at a level of detail so far inaccessible to conventional light optical techniques [15–17]. Among SRM, methods of Single Molecule Localization Microscopy (SMLM) received major attention as they probably require the least sophisticated instrumentation and provide very high resolution and a very good signal-to-background ratio [18]. The general principle of all SMLM approaches is to perform multiple registrations of the same fluorescently labeled object. In each frame, only a relatively small subset of fluorophore signals is detected, in such a way that these signals appear isolated on the detector; i.e. the minimum distance between the individual fluorescent sites is larger than the conventional resolution (for microscopes with high numerical aperture objective lenses about half a wavelength, or ca. 200 nm); the positions of these “optically isolated” fluorophore molecules are determined with nanometer precision and juxtaposed in a localization map. In most SMLM approaches, the recorded subset of fluorophores is reverted to a non-detectable state, and a different subset of fluorophores is acquired in the following frame. This process is repeated many times. Through combining the positions of the fluorophores obtained in a large number (thousands) of individual frames, a joint localization map (image) with a highly enhanced optical (smallest distance measured between two fluorophore sites) and structural resolution is obtained [19].

The chromatin distribution within the cell nucleus can be visualized through highlighting the DNA. This has been done in several ways but the most precise and efficient one employs DNA-binding dyes (intercalators or minor-groove binders) that may bind to the DNA double helix even every 3.2 nm^20^. Previous attempts that focused on SMLM of isolated DNA and small structures (such as the genome of prokaryotes) yielded spectacular results [21–23]. For example, isolated DNA of λ-phage was imaged with a resolution below 10 nm using Binding Activated Localization Microscopy (BALM) [23]. However, despite the excellent performance of the aforementioned techniques on small DNA structures, they remained rather cumbersome when applied to study the organization of chromatin within the nucleus of an eukaryotic cell [24,25]. In this article we will highlight a novel methodological solution.

## DNA properties provide a mechanism for fluorescence switching

SMLM is often implemented using fluorescent fusion proteins or immuno-labeling with organic fluorophores as reporter molecules. Control over the fluorescent properties of a molecular label and the potential for single molecule detection, i.e. optical isolation, is usually provided by stochastic reduction and oxidation of the fluorophore combined with appropriate laser frequencies and intensities (photoswitching [26–28]) or by altering the spectral properties via a change in the molecular structure (photoactivation and photoconversion [29]). Unfortunately, these mechanisms are rather ineffective when applied to fluorescent DNA-binding dyes; hence the need to search for alternative fluorescence switching solutions. In our recent publication [30] the fluorescence switching within the DNA-dye complex was conceived differently: instead of relying on the photophysical properties of permanently DNA-associated fluorescent dye directly, the properties of its binding partner, namely the DNA double helix were targeted.

Under physiological conditions DNA can adopt a set of different structural conformations among which the most stable is prevalent. This phenomenon is thermally driven and is known as “DNA breathing”. It is believed that “breathing” in physiological conditions serves as a mechanism which enables DNA-association of various proteins of enzymatic activity such as helicases [31]. In a physiological environment, DNA “breathing” is manifested in reversible and stochastic cycling within sub-milliseconds between conformational states of the native double-helix and some less frequent ones [31]. Under DNA-denaturing conditions, these latter states departing from the most stable conformation should become more abundant. In fact, strong denaturation procedures may lead to an irreversible collapse of the double helix and the formation of two single stranded DNA molecules [32,33].

Many DNA-binding dyes in aqueous solution are essentially non-fluorescent due to additional competitive decay channels of the excited state resulting from rapid intra-molecular dynamics [34]. However, upon binding to the DNA (irrespectively of the binding mechanism), some DNA-binding dyes experience a significant increase by a factor of 10³ or more in their fluorescence photon yield [34,35]. This increase in quantum efficiency requires, however, binding to a DNA site with stable local double stranded conformation (dsDNA). In its absence, e.g. when strong denaturing conditions are applied, the fluorescence of the DNA-binding dye is significantly reduced [36]. To summarize, the reversible transients between different DNA conformations in a carefully adjusted denaturing environment could be expected to indirectly enable fluorescence intensity switching at single DNA-binding site(s) in a controlled fashion.

## BALM in the cell nucleus

**Figure 1.**
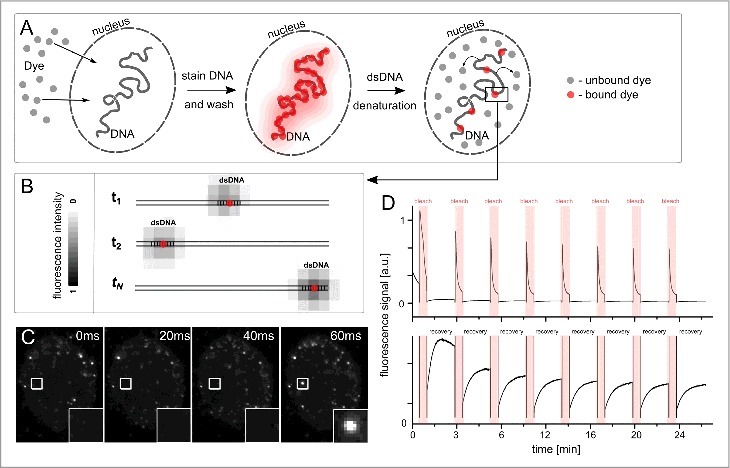
DNA structure fluctuation binding activated localization microscopy (fBALM). A) Scheme demonstrating the sample preparation protocol including nuclear membrane permeabilization and DNA-binding probe delivery followed by gradual pH decrease. B) Local DNA sequences undergoing stochastic conformational changes in a denaturing environment in fBALM and corresponding local fluorescence emission (pseudo-gray shades) of a DNA-binding dye (red dots) occurring upon sequence stabilization. The stochastic behavior of the DNA sequences is depicted for several time points (t_1_, t_2_,…,t_N_). C) Raw fBALM frames acquired using a CCD camera with a 20 ms exposure time. The small inset indicates a part of a cell nucleus that was stained with Sytox Orange where no signal appears prior the first 60 ms of the acquisition, corresponding to a denatured DNA site [36]. In the following frame a transiently fluorescent signal appears, as an underlying DNA sequence present at this site gains a stable double stranded conformation (inset for 60 ms frame). D) Time-course photobleaching experiment in fBALM conditions. The single cell nucleus shown in C was exposed repetitively to a high intensity exciting light (“bleach”, ∼1 kW/cm^2^, depicted in red) and low exciting light (∼20 W/cm^2^, no color) every ∼3 min. This was monitored by total nuclear fluorescence signal quantification (upper panel). Low intensity excitation fluorescence recovery profiles are enlarged and presented in the lower panel. The DNA-binding probe's fluorescence undergoes fast bleaching when exposed to high intensity excitation and recovers rapidly within only ∼2 min time period, suggesting the reversibility of the process underlying fluorescence switching, in a similar way as reported before [26]).

In our latest publication [30] we presented a novel single molecule-based method that advances DNA-BALM [23] to imaging the chromatin within the cell nucleus and enables studying nuclear architecture at an unprecedented level of structural resolution. This methodology termed as DNA structure fluctuation assisted BALM (fBALM) relies on a moderate nuclear membrane permeabilization followed by the delivery of a fluorescent DNA-binding dye inside the fixed cell nucleus (for scheme see Fig. 1A). Once cells are labeled and thoroughly washed, the pH in the tightly sealed microscopic sample is gradually decreased over the course of a few hours using a suitable imaging medium to slowly introduce local instabilities into the DNA structure. This takes place with simultaneous maintenance of native overall chromatin configuration as evaluated with a dedicated quantitative assay based on a chromatin-associated reference point-like signals (for details see [ref. 30]). In such an experimental setup, the pH eventually is lowered to a value of ∼3.7, i.e. a value that introduces reversible conformational changes to the dsDNA via ionization of the bases [33,37]. Due to a this instability introduced to the dsDNA, the fluorescence emission of the DNA-binding dye is rather transient as it can only originate efficiently from the DNA sequence that is stable (as presented in Fig. 1B). This is manifested well in single frames extracted from an unprocessed fBALM movie where bright flickering signals show up for tens to hundreds of milliseconds (Fig. 1C). These bright fluorescent signals correspond to local DNA sequences that underwent a transient conformational stabilization in a given moment (see inset in Fig. 1C). Such temporary fluorescence emissions from distinct locations in a sample and the thus achieved optical isolation are prerequisites for successful ‘blinking’ based super-resolution single molecule localization microscopy [15].

In Fig. 1D we demonstrate the behavior of a fluorescence signal of a Sytox Orange DNA-binding probe in the presence of cellular DNA exposed to 8 cycles of high and low intensity laser excitation light. The high intensity excitation cycle results in rapid signal decay whereas the low excitation cycle enables monitoring the signal recovery over a period of ∼2 min. We demonstrate that within approx. 24 min the DNA-binding dye signal recovers with an efficacy of ∼80% for 8 times pointing to adequate reversibility of the process [26].

**Figure 2.**
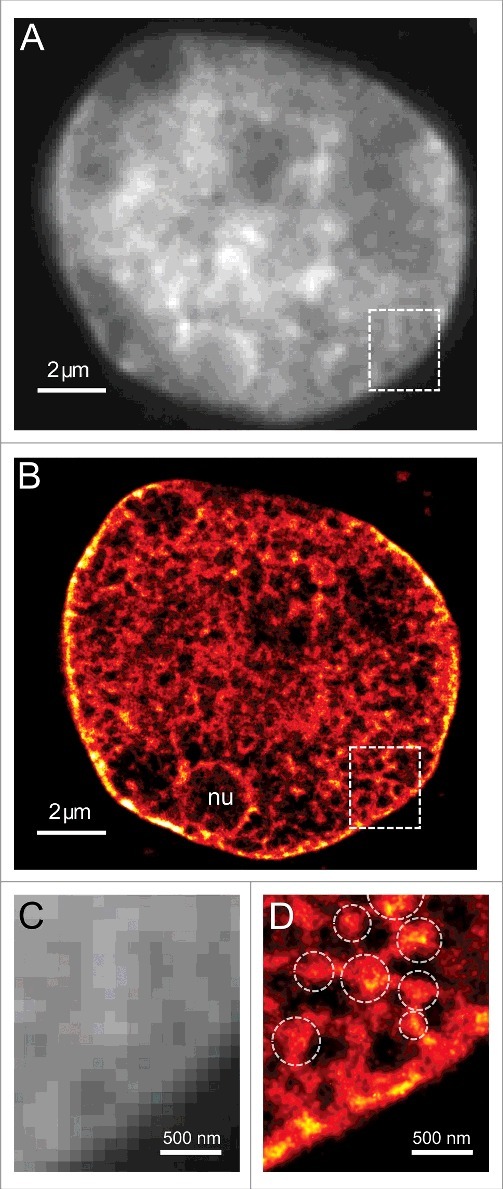
An example of an fBALM super-resolution image. A) Conventional image of the DNA labeled with YOYO-1^30^. B) fBALM image reconstructed based on SMLM principles of optical isolation. Nucleolus indicated with “nu”. C and D are zoomed-in insets of regions of interests embracing a part of the cell nucleus next to the nuclear periphery depicted in A and B, respectively. Domain-like chromatin organization becomes apparent due to enhanced resolution in fBALM (dense signal clusters are indicated with dashed circles in D). Vast parts of the cell nucleus indicate very low DNA densities only, in line with a previous interchromatin compartment model derived from 3D-SIM and electron microscopy images (reviewed in Cremer et al., 2015^1^). The single molecule localization density within the clusters reaches a value of 10,000/μm^2^, whereas at DNA-poor sites at most approx. some hundreds/μm^2^were detected.

The structural resolution in all SMLM techniques depends on the density of localized signals detected from the underlying fluorescently labeled structure and on the precision with which these signals can be localized [19]. In fBALM the number of signals available to capture is limited virtually only by the total acquisition time of the experiment (i.e. the total number of acquired frames) and this can be freely increased due to an efficient protection of the DNA-binding dye from photobleaching (Fig. 1D). Moreover, usage of small DNA-specific probes provides the best achievable labeling density among many other chromatin labeling techniques (compared in [30]). Furthermore, due to the relatively high number of photons emitted per event [38], the average localization precision inside the nucleus was ∼11 nm in the fBALM experiments reported. Thanks to these advantageous features of fBALM we were capable of discerning chromatin structures smaller than 50 nm^30^ (Fig. 2), outperforming significantly SMLM techniques of DNA-binding dye imaging in a cell nucleus based on in situ photoconversion [24,25]. Recently, similar attempts have been pursued using a custom designed fluorophore (Hoechst-JF_646_) and a SMLM technique termed point accumulation in nanoscale topography (PAINT) [19]. In fBALM, however, a number of conventional commercially available fluorophores can be used without the need for a complex chemical synthesis.

Different protein factors are known to occupy distinct compartments within the cell nucleus effectively determining its local functionality [1]. It would therefore be important to integrate fBALM with SRM of another spectrally distinguishable fluorescent probe to study the distribution of e.g. replication factories or DNA repair sites or FISH-labeled genomic loci with respect to the DNA density distribution. Presently, fBALM requires imaging in low pH; hence the combination with other photoswitchable fluorescent probes, which operate best at neutral pH values, is rather limited. As an alternative, fBALM may be combined with another super-resolution technique such as 3D Structured Illumination Microscopy (3D-SIM) [39]. Such a combination would enable imaging up to 2 other intra-nuclear targets with a resolution improved by a factor of 2 using SIM principles and produce an overlay with a high resolution DNA density map produced by fBALM (resolution improved by ∼5x).

## Future applications – future perspectives

In a first application of fBALM to study nuclear chromatin structure [30], we have demonstrated that in contrast to standard widefield microscopy it is capable of distinguishing quantitatively differences in the DNA density distribution in cells subjected to ischemic treatment: The chromatin of the cells deprived of oxygen and nutrients is known to bear decreased susceptibility to endonuclease digestion and decreased dynamic interaction of H1 histone [14]. Our fBALM experiments demonstrated that the chromatin in such ischemic conditions undergoes a vast condensation towards the nuclear periphery and that chromatin distribution in ischemia differs significantly from its native configuration [30]. The observed dramatic changes in chromatin configuration are largely reversible upon restoration of normal conditions (normoxia and normoglycemia).

Analysis of nuclear texture features as a measure of nuclear chromatin changes using conventional microscopy has been proven to be a highly effective tool to quantitatively describe changes in chromatin architecture associated with malignancy [40]. It is anticipated that the discrimination power of nuclear texture analysis will be substantially improved when nuclear chromatin structures are imaged at enhanced resolution. In line with this, another super-resolution microscopy technique termed 3D-SIM has already been employed to quantitatively assess differences in nuclear structure in Hodgkin's lymphoma [41,42], Multiple Myeloma (MM) [43,44], and in Alzheimer's disease [45], all obtained from patients. Whereas 3D-SIM of DAPI labeled DNA provides resolution in the order of 100 nm, fBALM could improve it further by another factor of ∼2 (i.e. enabling ∼ 50 nm structural resolution), increasing the precision of such chromatin texture studies taking advantage of already existent patients' sample preparation pipelines.

The application of 3D-SIM to cancer specimens has taught us a few important lessons; 1) cancer cells have a nuclear DNA architecture that is different from that of normal cells, and this difference can be quantified [41,42,44]. 2) Primary patient-derived cancer cells may be different depending on the stage of the disease. For example, myeloma cells of patients with monoclonal gammopathy of undetermined significance (MGUS), a precursor lesion to myeloma, show altered nuclear profiles of DNA organization compared to normal lymphocytes and to multiple myeloma [44]. It is of note that the amount of DNA-poor/free space increases from normal to MGUS, and from MGUS to MM. Similarly, its level significantly increases in Hodgkin's lymphoma (HL) from normal lymphocyte to mono-nucleated Hodgkin's (H) cell, and from H cells to bi-, tri- and tetra-nucleated Reed-Sternberg (RS) cell [42]. Importantly, bi-nucleated RS cells have less DNA-poor or DNA-free spaces than tri-nucleated RS cells, and tri-nucleated have less than tetra-nucleated RS cells. Thus, as HL progresses from H to RS cell and as RS cells increase their multinuclearity, DNA-poor/free space is increased.

These findings support an essential aspect of the interchromatin domain model [1] rather than the interchromosomal network model (reviewed in Branco & Pombo, 2006 [46]). The former model predicts a nuclear chromatin structure consisting of highly condensed inactive nuclear compartments (INC) as well as active nuclear compartments (ANC) with a substantially lower DNA content. This basic nanostructural feature is apparent in both SIM and fBALM images. In the interchromosomal network model, a much more homogeneous DNA distribution is expected, due to the high degree of intermingling assumed. Further, the ANC-INC model's assumptions remain in line with our observations on the reversible increase in interchromosomal space after ischemic stress [14,30]; again, a feature not assumed in the interchromosomal network model. Interestingly, recent 3D-genome reconstructions based on single cell Hi-C data demonstrated the spatial segregation of chromatin into A- and B-compartments [10]. It turns out that the genome's B-compartment localizes to the nuclear periphery and surrounds the nucleoli. With the help of fBALM we demonstrated that these chromatin regions bear a high DNA-density (Fig. 2B).

The clinically-relevant data summarized above are considered first insights into multiple possible medical applications that super-resolution technologies may provide us with in the foreseeable future. Thinking in terms of a near future for these technologies, one may easily imagine the value of these novel approaches for the exact staging of a patient's level of genomic instability, for the exact analysis of his/her tumor cell heterogeneity, and one may even consider the multi-faceted super-resolution applications for both diagnosis and monitoring, including their use as companion diagnostic tools. To achieve this future goal, larger cohort studies are required.

Current limitations to this powerful application include its computer-intensive and time-consuming image acquisition and analysis. It is, however, feasible to develop faster and more automated approaches to this powerful technology [47]. Only when these steps are achieved, can one think of future clinical applications using super-resolution as a tool to define structural biomarkers of disease.
